# Supplementary material for: Colorectal cancer trends in Chile: A Latin-American country with marked socioeconomic inequities
Source: PLoS One. 2022 Nov 10;17(11):e0271929. doi: 10.1371/journal.pone.0271929 (PMC9648833; doi:10.1371/journal.pone.0271929)
Supplement: S1 Appendix — (DOCX) [file pone.0271929.s001.docx]

S1 Appendix - FONASA groups

|  | **Income level (*)** | **Insurance coverage** |
| --- | --- | --- |
| Group A | No income or immigrants | 100% coverage in the public network |
|  | Family subsidy (Law 18.020) |  |
|  |  |  |
| Group B | Up to 319.000 CLP monthly | 100% coverage in the public network |
|  |  | Access to limited private services with copay |
|  |  |  |
| Group C | Between 319.000 and 465.740 CLP monthly | 90% coverage in the public network |
|  | With three or more dependents qualify to level B | Access to limited private services with copay |
|  |  |  |
| Group D | More than 465.740 CLP monthly | 80% coverage in the public network |
|  | With three or more dependents qualify to level C | Access to limited private services with copay |
| (*) 1 USD = 810 CLP | |  |

Table 1. Description of FONASA Groups.
