# Supplementary material for: Colorectal cancer trends in Chile: A Latin-American country with marked socioeconomic inequities
Source: PLoS One. 2022 Nov 10;17(11):e0271929. doi: 10.1371/journal.pone.0271929 (PMC9648833; doi:10.1371/journal.pone.0271929)
Supplement: S2 Appendix — (DOCX) [file pone.0271929.s002.docx]

# S2 Appendix - CRC ICD-10 diagnosis codes

| PRINCIPAL COLORECTAL CANCER DEATH and TREATMENT ICD-10 DIAGNOSIS CODES & DESCRIPTIONS | | | |
| --- | --- | --- | --- |
| C180 | Malignant neoplasm of cecum | D010 | Carcinoma in situ of colon |
| C182 | Malignant neoplasm of ascending colon | D011 | Carcinoma in situ of rectosigmoid junction |
| C183 | Malignant neoplasm of hepatic flexure | D012 | Carcinoma in situ of rectum |
| C184 | Malignant neoplasm of transverse colon | D374 | Neoplasm of uncertain behavior of colon |
| C185 | Malignant neoplasm of splenic flexure | D375 | Neoplasm of uncertain behavior of rectum |
| C186 | Malignant neoplasm of descending colon |  |  |
| C187 | Malignant neoplasm of sigmoid colon |  |  |
| C189 | Malignant neoplasm of colon, unspecified |  |  |
| C19X | Malignant neoplasm of rectosigmoid junction |  |  |
| C20X | Malignant neoplasm of rectum |  |  |

Table 2. Principal CRC deaths and discharges ICD-10 diagnosis codes & descriptions.

| ADDITIONAL PRINCIPAL COLORECTAL CANCER DEATH and RELATED TREATMENT ICD-10 DIAGNOSIS CODES & DESCRIPTIONS | | | |
| --- | --- | --- | --- |
| D120 | Benign neoplasm of cecum | D125 | Benign neoplasm of sigmoid colon |
| D122 | Benign neoplasm of ascending colon | D126 | Benign neoplasm of colon, unspecified |
| D123 | Benign neoplasm of transverse colon | D127 | Benign neoplasm of rectosigmoid junction |

Table 3. Additional Principal CRC deaths and Related CRC discharges ICD-10 diagnosis codes & descriptions.

| RELATED COLORECTAL CANCER DEATH and TREATMENT ICD-10 DIAGNOSIS CODES & DESCRIPTIONS | | | |
| --- | --- | --- | --- |
| C181 | Malignant neoplasm of appendix | K565 | Other impaction of intestine |
| C188 | Malignant neoplasm of overlapping sites of colon | K566 | Intestinal adhesions [bands] with complete obstruction |
| C210 | Malignant neoplasm of anus, unspecified | K621 | Rectal polyp |
| C211 | Malignant neoplasm of anal canal | K624 | Stenosis of anus and rectum |
| C218 | Malignant neoplasm of overlapping sites of rectum, anus and anal canal | K625 | Hemorrhage of anus and rectum |
| C227 | Other specified carcinomas of liver | K626 | Ulcer of anus and rectum |
| C229 | Malignant neoplasm of liver, not specified as primary or secondary | K629 | Disease of anus and rectum, unspecified |
| C260 | Malignant neoplasm of intestinal tract, part unspecified | K630 | Abscess of intestine |
| C268 | Malignant neoplasm of spleen | K631 | Perforation of intestine (nontraumatic) |
| C269 | Malignant neoplasm of ill-defined sites within the digestive system | K639 | Disease of intestine, unspecified |
| C480 | Malignant melanoma of skin, unspecified | K914 | Postprocedural complete intestinal obstruction |
| C762 | Malignant neoplasm of endocrine gland, unspecified | K922 | Gastrointestinal hemorrhage, unspecified |
| C785 | Malignant neoplasm of endocrine gland, unspecified | Z031 | Encounter for administrative examinations, unspecified |
| C786 | Malignant neoplasm of endocrine gland, unspecified | Z080 | Encounter for follow-up examination after completed treatment for malignant neoplasm |
| C787 | Malignant neoplasm of endocrine gland, unspecified | Z081 | Encounter for follow-up examination after completed treatment for malignant neoplasm |
| C809 | Malignant neoplasm associated with transplanted organ | Z082 | Encounter for follow-up examination after completed treatment for malignant neoplasm |
| C80X | Malignant neoplasm associated with transplanted organ | Z087 | Encounter for follow-up examination after completed treatment for malignant neoplasm |
| C97X | Malignant neoplasm of lymphoid, hematopoietic and related tissue, unspecified | Z088 | Encounter for follow-up examination after completed treatment for malignant neoplasm |
| D013 | Carcinoma in situ of anus and anal canal | Z089 | Encounter for follow-up examination after completed treatment for malignant neoplasm |
| D019 | Carcinoma in situ of digestive organ, unspecified | Z432 | Encounter for attention to ileostomy |
| D097 | Carcinoma in situ of thyroid and other endocrine glands | Z433 | Encounter for attention to colostomy |
| D377 | Benign neoplasm, unspecified site | Z510 | Encounter for antineoplastic radiation therapy |
| D379 | Benign neoplasm, unspecified site | Z511 | Encounter for antineoplastic radiation therapy |
| D489 | Neoplasm of uncertain behavior, unspecified | Z512 | Encounter for antineoplastic immunotherapy |
| D630 | Anemia in neoplastic disease | Z932 | Ileostomy status |
| K564 | Gallstone ileus | Z933 | Colostomy status |

Table 4. Related CRC deaths and related discharges ICD-10 diagnosis codes & descriptions.
