# Supplementary material for: Colorectal cancer trends in Chile: A Latin-American country with marked socioeconomic inequities
Source: PLoS One. 2022 Nov 10;17(11):e0271929. doi: 10.1371/journal.pone.0271929 (PMC9648833; doi:10.1371/journal.pone.0271929)
Supplement: S3 Appendix — (DOCX) [file pone.0271929.s003.docx]

# S3 Appendix - ICD-10 discharge codes for CRC-deceased patients without CRC-related discharges

| ICD-10 DIAGNOSIS CODES, DESCRIPTIONS & NUMBER OF PATIENTS | | |
| --- | --- | --- |
| J189 | Pneumonia, unspecified organism | 453 |
| A419 | Sepsis, unspecified organism | 235 |
| N390 | Urinary tract infection, site not specified | 198 |
| N40X | Nodular prostate with lower urinary tract symptoms | 190 |
| D649 | Anemia, unspecified | 186 |
| K802 | Calculus of gallbladder with other cholecystitis with obstruction | 175 |
| H269 | Unspecified cataract | 170 |
| K409 | Unilateral inguinal hernia, with gangrene, recurrent | 154 |
| S720 | Open bite, unspecified thigh, sequela | 147 |
| N10X | Acute pyelonephritis | 128 |
| I64X | Cerebral infarction, unspecified | 92 |
| I802 | Phlebitis and thrombophlebitis of femoral vein, bilateral | 87 |
| N189 | Chronic kidney disease, unspecified | 84 |
| I509 | Heart failure, unspecified | 82 |
| J449 | Chronic obstructive pulmonary disease, unspecified | 81 |
| I678 | Cerebral arteritis, not elsewhere classified | 79 |
| K579 | Diverticulitis of intestine, part unspecified, with perforation and abscess with bleeding | 79 |
| K810 | Acute cholecystitis | 78 |
| L031 | Acute lymphangitis of unspecified toe | 73 |
| I500 | Cardiac arrhythmia, unspecified | 73 |
| K439 | Ventral hernia without obstruction or gangrene | 68 |
| K805 | Calculus of bile duct with acute and chronic cholecystitis with obstruction | 67 |
| I219 | Acute myocardial infarction, unspecified | 66 |
| C169 | Malignant neoplasm of stomach, unspecified | 66 |
| C786 | Malignant neoplasm of endocrine gland, unspecified | 66 |
| R104 | Periumbilical pain | 65 |
| H259 | Unspecified age-related cataract | 63 |
| I10X | Essential (primary) hypertension | 62 |
| I200 | Unstable angina | 61 |
| A09X | Infectious gastroenteritis and colitis, unspecified | 60 |

Table 5. Top thirty ICD-10 discharge codes for CRC-deceased patients without CRC-related discharges. Each patient is counted at most once per code but might be counted in multiple different codes if applicable.
