# Supplementary material for: Colorectal cancer trends in Chile: A Latin-American country with marked socioeconomic inequities
Source: PLoS One. 2022 Nov 10;17(11):e0271929. doi: 10.1371/journal.pone.0271929 (PMC9648833; doi:10.1371/journal.pone.0271929)
Supplement: S4 Appendix — (DOCX) [file pone.0271929.s004.docx]

S4 Appendix - Geography of Chile: regions and public hospitals


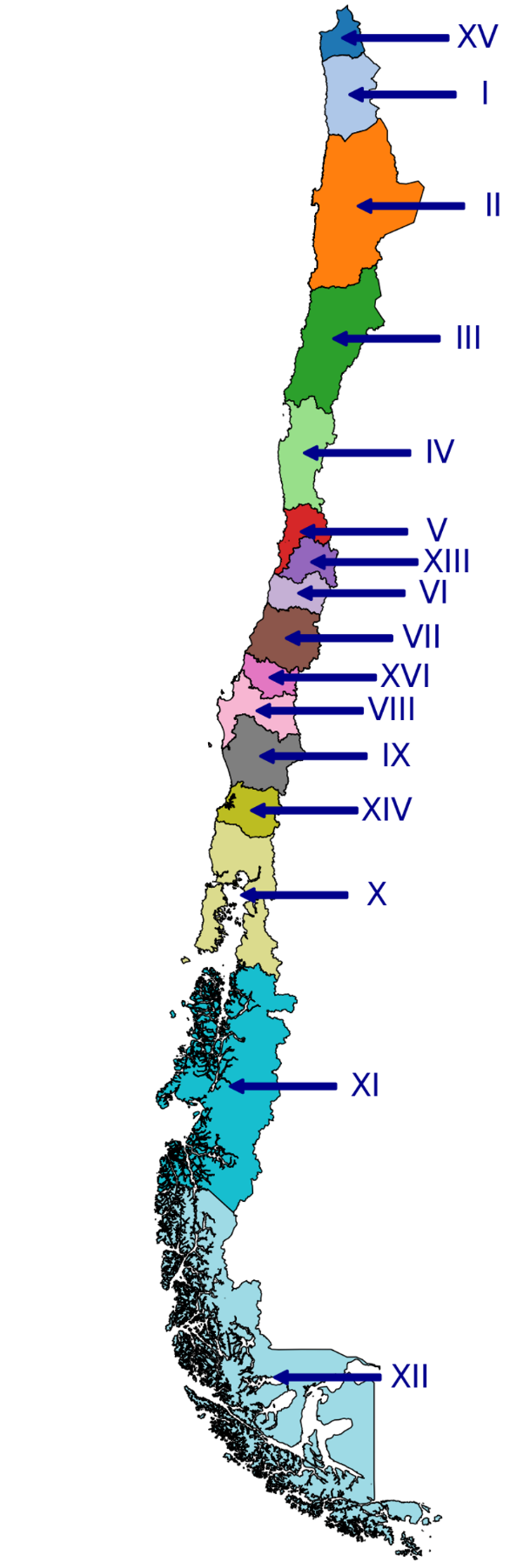

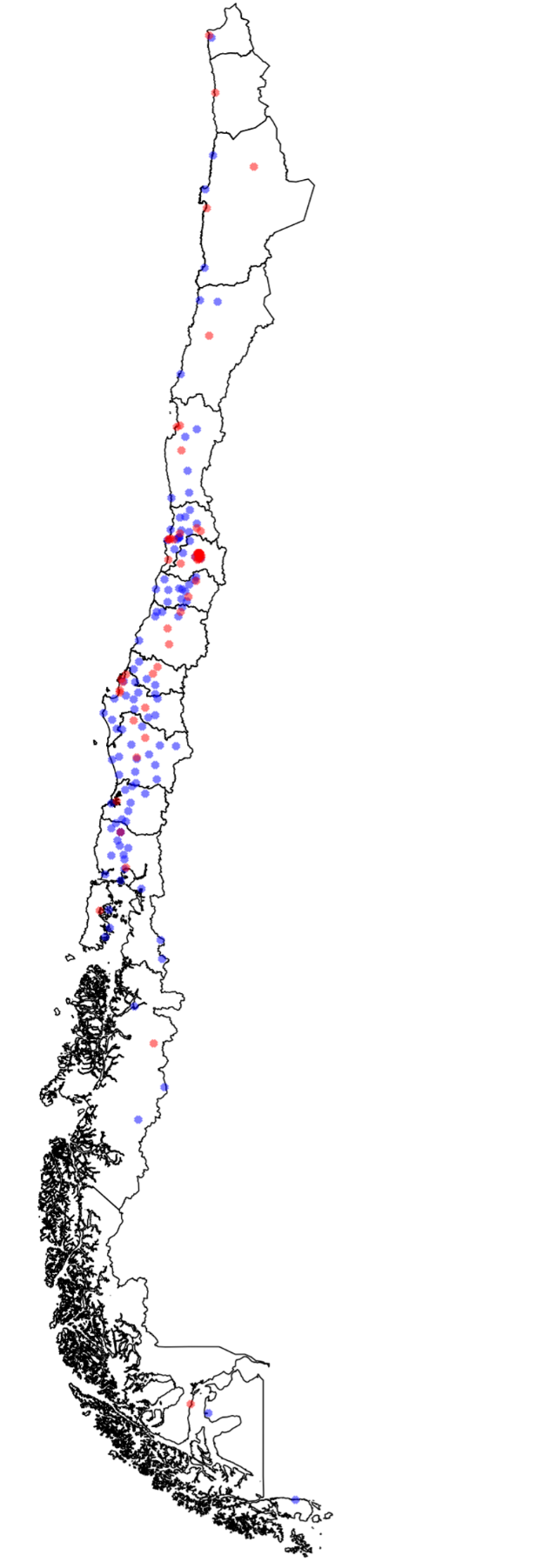


Fig 1: Left: Geopolitical map of continental Chile, with its fifteen regions. Right: Locations of public hospitals, with low and medium complexity centers in blue, and high complexity in red. Map source: prepared by the authors from public domain polygonal data released by the Chilean National Library of Congress.
