# Supplementary material for: Colorectal cancer trends in Chile: A Latin-American country with marked socioeconomic inequities
Source: PLoS One. 2022 Nov 10;17(11):e0271929. doi: 10.1371/journal.pone.0271929 (PMC9648833; doi:10.1371/journal.pone.0271929)
Supplement: Attachment — Submitted filename: PONE-D-22-00874.docx [file pone.0271929.s005.docx]

This paper analyzed the trends in the last ten years and the influence of observable factors on survival, including explicit guarantees in CRC treatment access (GES program).

Depending on socioeconomic status, survival was significantly better with private insurance than the national insurance system. Patients in the capital city survived longer than those in other parts of the

Replenish persuasively in discussions. Note the following:

Kim, J.I., Kim, G. Effects on inequality in life expectancy from a social ecology perspective. BMC Public Health 18, 243 (2018). https://doi.org/10.1186/s12889-018-5134-1
